# Motif-pattern dependence of biomolecular phase separation driven by specific interactions

**DOI:** 10.1371/journal.pcbi.1009748

**Published:** 2021-12-29

**Authors:** Benjamin G. Weiner, Andrew G. T. Pyo, Yigal Meir, Ned S. Wingreen

**Affiliations:** 1 Department of Physics, Princeton University, Princeton, New Jersey, United States of America; 2 Department of Physics, Ben Gurion University of the Negev, Beer-Sheva, Israel; 3 Department of Molecular Biology, Princeton University, Princeton, New Jersey, United States of America; 4 Lewis-Sigler Institute for Integrative Genomics, Princeton University, Princeton, New Jersey, United States of America; Heidelberg Institute for Theoretical Studies (HITS gGmbH), GERMANY

## Abstract

Eukaryotic cells partition a wide variety of important materials and processes into biomolecular condensates—phase-separated droplets that lack a membrane. In addition to nonspecific electrostatic or hydrophobic interactions, phase separation also depends on specific binding motifs that link together constituent molecules. Nevertheless, few rules have been established for how these ubiquitous specific, saturating, motif-motif interactions drive phase separation. By integrating Monte Carlo simulations of lattice-polymers with mean-field theory, we show that the sequence of heterotypic binding motifs strongly affects a polymer’s ability to phase separate, influencing both phase boundaries and condensate properties (e.g. viscosity and polymer diffusion). We find that sequences with large blocks of single motifs typically form more inter-polymer bonds, which promotes phase separation. Notably, the sequence of binding motifs influences phase separation primarily by determining the conformational entropy of self-bonding by single polymers. This contrasts with systems where the molecular architecture primarily affects the energy of the dense phase, providing a new entropy-based mechanism for the biological control of phase separation.

## Author summary

Cells need to concentrate biomolecules in the right place at the right time in order to function. Many important intracellular compartments are liquid droplets formed by phase separation, the same process that separates oil from vinegar. The properties of such “biomolecular condensates” depend on the component molecules, such as proteins and RNAs. These molecules are polymers made of many interacting monomers, often organized into “motifs,” and the sequence of motifs shapes the properties of the condensates. Recent work has revealed important principles governing phase separation when the motifs are charged and interact across long distances, but many phase-separating molecules form specific interactions that are short-range and one-to-one. How does the sequence of specifically-interacting motifs affect phase separation? Using a combination of simulations and theoretical calculations, we show that the sequence has profound effects on both the formation and properties of condensates. Sequences with large blocks of identical motifs are better at phase separating but more viscous and solid-like. Importantly, we find that sequence controls phase separation via the proclivity to form self-bonds instead of forming bonds with other polymers. Thus the sequence of specifically-interacting motifs provides a control point for the formation and properties of phase-separated intracellular compartments.

## 1 Introduction

Understanding how biological systems self-organize across spatial scales is one of the most pressing questions in the physics of living matter. It has recently been established that eukaryotic cells use phase-separated biomolecular condensates to organize a variety of intracellular processes ranging from ribosome assembly and metabolism to signaling and stress response [1–3]. Biomolecular condensates are also thought to play a key role in physically organizing the genome and regulating gene activity [4–6]. How do the properties of these condensates emerge from their components, and how do cells regulate condensate formation and function? Unlike the droplets of simple molecules or homopolymers, intracellular condensates are typically composed of hundreds of molecular species, each with multiple interaction motifs. These interaction motifs can include folded domains, such as in the nephrin-Nck-N-WASP system for actin regulation [7], or individual amino acids in proteins with large intrinsically disordered regions (IDRs), such as the germ granule protein Ddx4 [8]. While the precise sequences of these motifs are believed to play a major role in determining condensates’ phase diagrams and material properties, the nature of this relation has only begun to be explored [9–11]. As a result, it remains difficult to predict the formation, properties, and composition of these diverse functional compartments.

Previous studies have established important principles relating phase separation to the sequence of nonspecific interaction domains such as electrostatic or hydrophobic motifs. For example, polyampholytes (polymers with charged monomers) have been studied using random-phase approximation (RPA) theory [12, 13], field-theoretic simulations [14], lattice simulations [15], molecular dynamics simulations [16, 17], and experiments [18]. A common theme is that the sequence of charges has a strong effect on phase separation: large blocks of like charge promote condensation by making the dense phase more energetically favorable. In the case of polyelectrolytes (multicomponent systems where each polymer is highly positive or negative overall), the entropy associated with counterion condensation also plays a major role in modulating sequence-dependent phase separation [19]. For hydrophobic interactions, sequence controls the structure of the dense phase: liquids, structured liquids, and aggregates such as micelles and membranes can all appear depending on the sequence of hydrophobic and hydrophilic residues [20]. Several studies have also noted correlations between single-polymer properties, such as the radius of gyration and theta-temperature, and thermodynamic properties, such as the critical temperature [21, 22], raising the intriguing possibility that the sequence-dependence of complicated many-polymer interactions can be explained by simpler self-interactions.

However, in many cases condensate formation and function depend on specific interactions which are short-range, one-to-one, and saturating [2]. We expect these to obey different physical principles than electrostatic or hydrophobic interactions. For example, a charged monomer interacts with all its neighbors, whereas a specific-interaction motif can form only a single bond, reducing the energetic drive to aggregate. Such one-to-one interactions between heterotypic domains are ubiquitous in biology, and they include residue-residue bonds, bonds between protein domains, protein-RNA bonds, and RNA-RNA bonds. Recent studies have enumerated a large number of examples in both one-component [23] and two-component [24, 25] systems (e.g. cation-pi bonds between tyrosine and arginine in FUS-family proteins, bonds between protein domains in the SIM-SUMO system). Another important example is RNA phase separation in “repeat-expansion disorders” such as Huntington’s disease and ALS. There, phase separation is driven by specific interactions between nucleotides arranged into regular repeating blocks, and it has recently been shown that the repeated sequence pattern is necessary for aggregate formation [26]. In spite of the biological importance of such specific interactions, their statistical mechanical description remains undeveloped. Here, we address the important question: what is the role played by sequence when specific, heterotypic interactions are the dominant drivers of phase separation?

Specifically, we analyzed a novel model of polymers with specific, heterotypic interaction motifs using Monte Carlo simulations and mean-field theory. Our use of advanced Monte Carlo techniques allowed us to rigorously determine thermodynamic properties such as the critical point and binodal curves. We then developed a mean-field theory linking single-polymer behavior obtainable from short simulations to emergent phase behavior. This integration of theory and simulation allowed us to uncover clear sequence design principles which would be difficult to discern from either approach on its own. Importantly, our mixed approach captures strong correlations in self-interactions which are neglected by RPA [13]. We found that motif sequence determines both the size of the two-phase region and dense-phase properties such as viscosity and polymer extension. Importantly, sequence acts primarily by controlling the entropy of self-bonds. This suggests a new paradigm for biological control of intracellular phase separation: when bonds are specific and saturating, the entropy of *intra*molecular interactions can be just as relevant as the energy of *inter*molecular interactions.

## 2 Results

**Fig 1:**
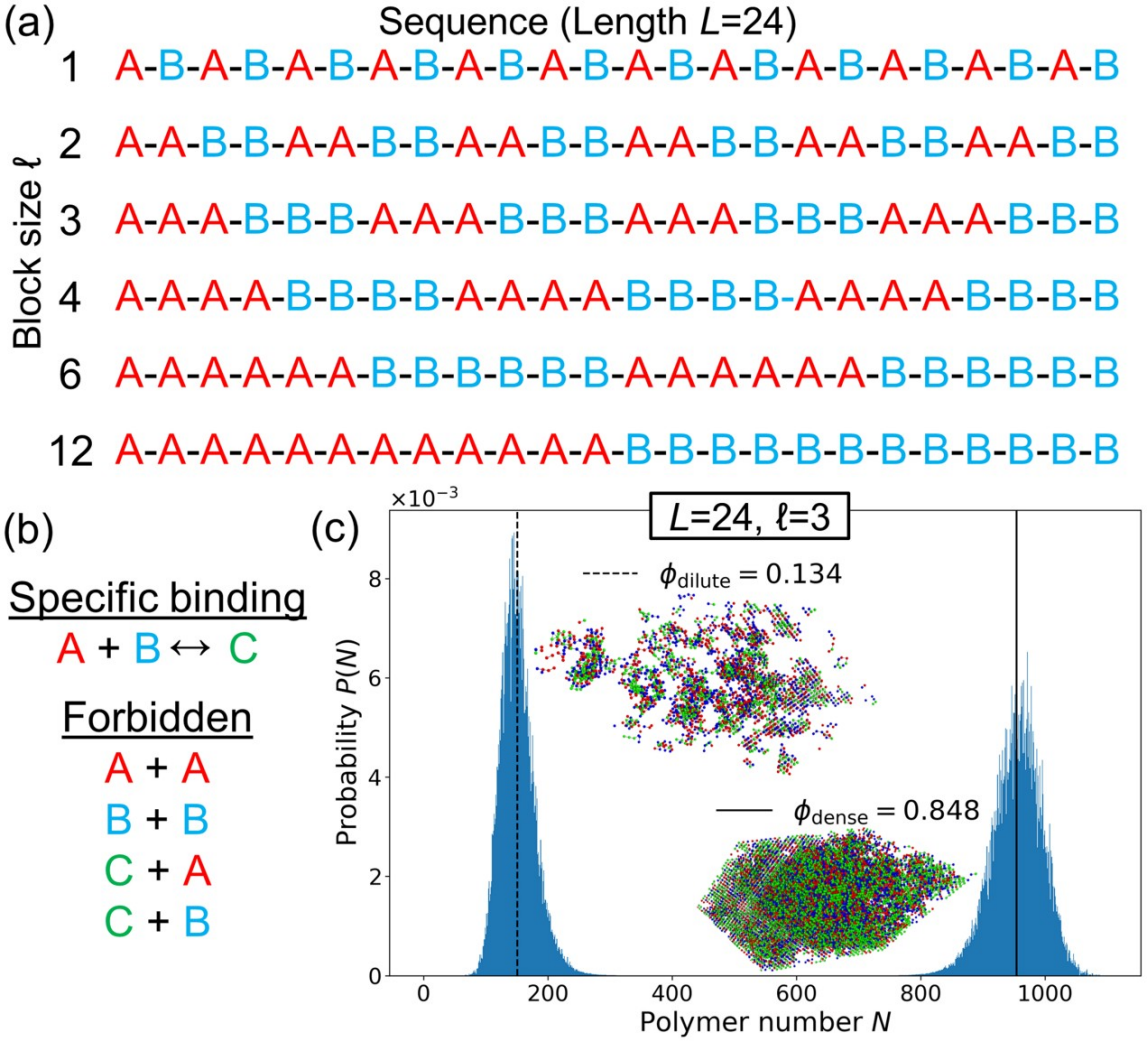
Lattice model for phase separation by polymers with one-to-one interacting motifs. (a) Each polymer is defined by its sequence of motifs, which come in types “A” (red) and “B” (blue). The class of sequences shown consists of repeated blocks of As and Bs, labeled by their block size *ℓ*. (b) In lattice simulations, an A and a B motif on the same lattice site form a specific, saturating bond (green) with binding energy *ϵ*. Monomers of any type on adjacent lattice sites have an attractive nonspecific interaction energy *J* = 0.05*ϵ*. A-A and B-B overlaps are forbidden. (c) Polymer number distribution *P*(*N*) at the phase boundary of the *ℓ* = 3 sequence (*βϵ* = 0.9287, *μ* = −9.9225*ϵ*). At fixed *μ* the system fluctuates between two phases. *Inset*: Snapshots of the GCE (fixed *μ*) simulation at *ϕ*_dilute_ and *ϕ*_dense_.

How does a polymer’s sequence of interaction motifs affect its ability to phase separate? To address this question, we developed a lattice model where each polymer consists of a sequence of “A” and “B” motifs which form specific, saturating bonds of energy *ϵ* (Fig 1a and 1b). We used the three-dimensional FCC lattice because it is the Bravais lattice with the highest coordination number (12 neighbors, as opposed to 6 on a cubic lattice), most closely mimicking free space. (Although the restriction to a lattice limits polymer conformations, we expect this effect to be sequence-independent, allowing us to compare results across sequences.) A bond forms when an “A” and a “B” monomer occupy the same lattice site, reflecting the reduced volume of bonds (e.g. when a cation is held in an aromatic ring or folded protein domains fit closely together.) Monomers on adjacent lattice sites also have nonspecific interaction energy *J*. For each sequence, we determined the phase diagram, which describes the temperatures and polymer concentrations at which droplets form. To enable full characterization of the phase diagram including the critical point, we used Monte Carlo simulations in the Grand Canonical Ensemble (GCE), where the number of polymers *N* in the simulation can fluctuate: the 3D conformations of the polymers are updated using a predefined move-set, and polymers are inserted/deleted with chemical potential *μ*. (See Methods and materials for details.) For each sequence, we determined the critical point (temperature *T*_c_ and chemical potential *μ*_c_). Then for each *T* < *T*_c_ we located the phase boundary, defined by the value μ* for which the dilute and dense phases have equal thermodynamic weight. Around this value of *μ*, the system transitions back and forth between the two phases throughout the simulation, leading to a polymer number distribution *P*(*N*) that has two peaks with equal weights (Fig 1c) [27]. The dilute and dense phase concentrations *ϕ*_dilute_ and *ϕ*_dense_ are the means of these two peaks. Multicanonical sampling was employed to adequately sample transitions (Methods and materials).

**Fig 2:**
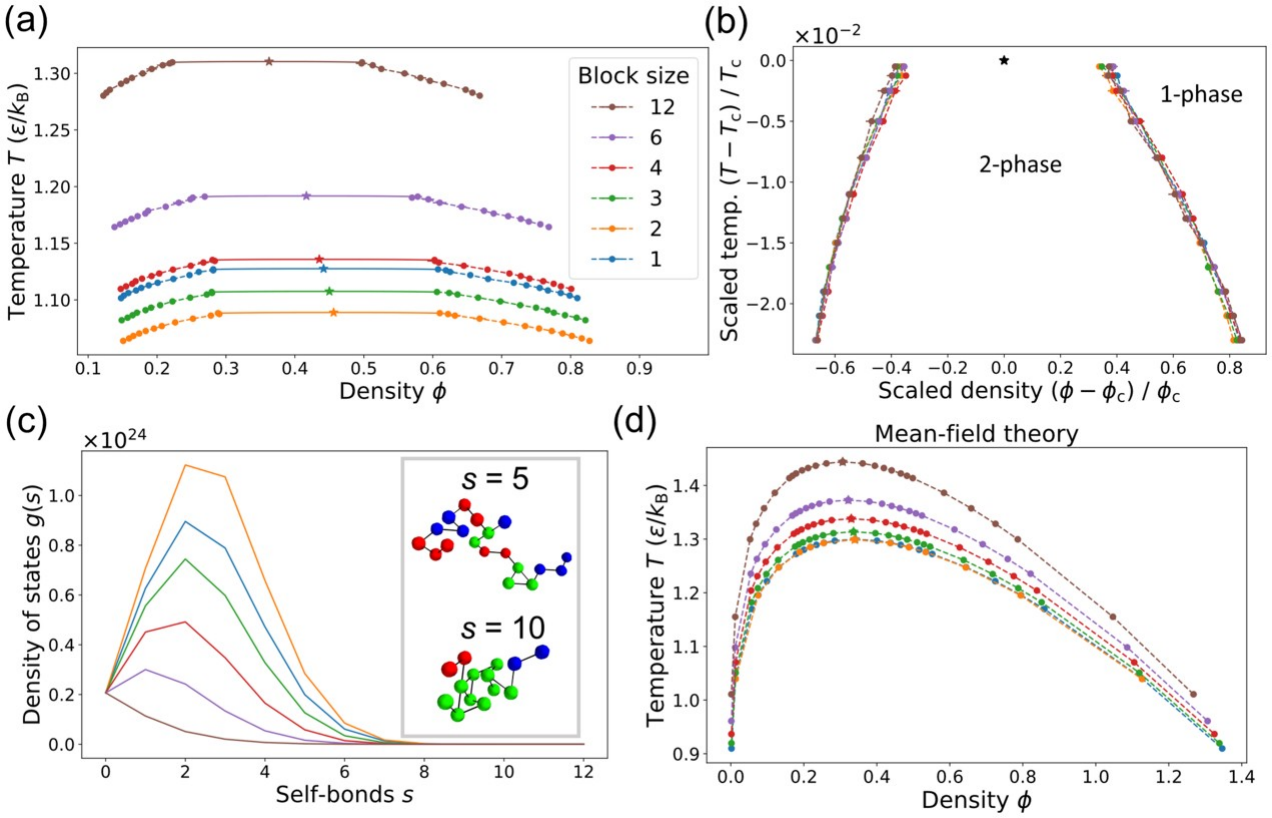
The sequence of binding motifs strongly affects a polymer’s ability to phase separate. (a) Binodal curves defining the two-phase region for the six sequences of length *L* = 24 shown in Fig 1a. Stars indicate the critical points and the solid curves are fits to scaling relations for the 3D Ising universality class. Mean ± SD for three replicates. (Uncertainties are too small to see for most points.) Color key applies to all panels. (b) When rescaled by the critical temperature *T*_c_ and critical density *ϕ*_c_, the phase boundaries in (a) collapse, even far from the critical point. (c) The tendency to phase separate is inversely related to the density of states *g*(*s*), i.e. the number of ways a given sequence can form *s* bonds with itself. Inset: Snapshots of *ℓ* = 3 polymer with *s* = 5 (top) and *s* = 10 (bottom). Black lines show the polymer backbone. (d) Phase boundaries from mean-field theory using *g*(*s*) (Eq 1).

We first constructed phase diagrams for polymers with the six sequences shown in Fig 1a, all with *L* = 24 motifs arranged in repeating blocks, and all with equal numbers of A motifs and B motifs (*a* = *b* = 12 where *a* and *b* are the numbers of A and B motifs in a sequence). Each simulation contains polymers of a single sequence, and the sequences differ only in their block sizes *ℓ*. Fig 2a shows the resulting phase diagrams, which differ dramatically by block size, e.g. the *T*_c_ values for *ℓ* = 2 and *ℓ* = 12 differ by 20%. The absolute magnitude of the effect depends on the interaction energy scale *ϵ*, but we note that if the *T*_c_ for *ℓ* = 12 were in the physiological range around 300K, the corresponding 60K difference would render the condensed phase of *ℓ* = 2 inaccessible in most biological contexts. Despite this wide variation, Fig 2b shows that rescaling by *T*_c_ and *ϕ*_c_ causes the curves to collapse. This is expected near the critical point, where all sequences share the behavior of the 3D Ising universality class [27], but the continued nearly exact data collapse indicates that (*T*_c_, *ϕ*_c_) fully captures the sequence-dependence of the phase diagram.

Why does the sequence of binding motifs have such a strong effect on phase separation? Importantly, sequence determines the entropy of intra-polymer bonds, i.e. the facility of a polymer to form bonds with itself. This is quantified by the single-polymer density of states *g*(*s*): for each sequence, *g*(*s*) counts the number of 3D conformations with *s* self-bonds. For short polymers, *g*(*s*) can be enumerated, whereas for longer polymers, it can be extracted from Monte Carlo simulations of a single polymer (see Methods and materials for details). This procedure captures strong correlations between intrachain bonds (each bond excludes other pairings) which are neglected by RPA but important for determining the entropy of self-interactions. Fig 2c shows *g*(*s*) for each of the block sequences, obtained from Monte Carlo simulations. Sequences with small block sizes have many more conformations available to them at all values of *s*. Intuitively, a sequence like *ℓ* = 2 allows a polymer to make many local bonds, whereas a sequence like *ℓ* = 12 cannot form multiple bonds without folding up globally like a hairpin. Such hairpin states are thermodynamically unfavorable at these temperatures due to the low conformational entropy, so it is more favorable for polymers like *ℓ* = 12 to phase separate and form trans-bonds with others, leading to a high *T*_c_ value. Even when *T* < *T*_c_ so that low-energy states with many bonds are favored, large-block sequences have large two-phase regions because *g*(*s*) is small for all *s*. Thus, polymers with large blocks form condensates over a much wider range of temperatures and concentrations.

This intuition can be captured by a simple mean-field theory that incorporates only single-polymer properties, namely *g*(*s*) and the number of A and B motifs per polymer, *a* and *b*. We calculate the free energy density of a state where each polymer forms *s* self-bonds and *t* trans-bonds (bonds with other polymers). We make two mean-field simplifications: 1) every polymer has the mean number of trans-bonds $\bar{t}$, and 2) each polymer interacts with the others through a mean-field background of independent motifs. In contrast, the self-interaction is described by the full density of states *g*(*s*) extracted from single-polymer simulations. This leads to the following free energy density (see “Mean-field theory” in S1 Text for derivation):

$$\begin{array}{c} f\left(\bar{s},\bar{t}\right)\equiv \frac{F}{k_{B}TV}=f_{\text{steric}}\left(\bar{s},\bar{t}\right)+f_{\text{trans}}\left(\bar{s},\bar{t}\right)+\beta \chi \phi^{2}-\frac{\phi }{L}(\;\text{log}\;\sum_{s}g\left(s\right)e^{ws})+\frac{\phi }{L}w\bar{s}-\frac{\phi }{L}\beta \epsilon (\bar{s}+\frac{\bar{t}}{2}), \end{array}$$ (1)

where *V* is the number of lattice sites, *χ* is the nonspecific-interaction parameter,

$$\begin{array}{c} f_{\text{steric}}\equiv \frac{\phi }{L}\;\text{log}\;\frac{\phi }{L}+(1-\phi \frac{\left\langle l\right\rangle }{L})\;\text{log}\;(1-\phi \frac{\left\langle l\right\rangle }{L})+\frac{\phi }{L}(\langle l\rangle -1),\;\left\langle l\right\rangle \equiv L-\bar{s}-\bar{t}/2, \end{array}$$ (2)

and

$$\begin{array}{c} \begin{array}{cc} f_{\text{trans}} & \equiv \frac{\phi }{L}(y\left(a\right)+y\left(b\right)+\frac{\bar{t}}{2}\;\text{log}\;\frac{\bar{t}}{2}+\frac{\bar{t}}{2}(1-\;\text{log}\;\frac{\phi }{L})),\\ y(x) & \equiv \left(x-\bar{s}-\bar{t}/2\right)\;\text{log}\;\left(x-\bar{s}-\bar{t}/2\right)-\left(x-\bar{s}\right)\;\text{log}\;\left(x-\bar{s}\right). \end{array} \end{array}$$ (3)

*f*_steric_ is the translational contribution from the number of ways to place polymers without overlap and *f*_trans_ is the entropy of forming $\bar{t}$ trans-bonds given $\bar{s}$ self-bonds, derived from the combinatorics of pairing independent motifs. The fourth term in Eq 1 accounts for the self-bonding entropy, where *w* is the self-bond weight chosen to self-consistently enforce $\sum_{i}s_{i}/N=\bar{s}$. The next term is the Legendre transform compensating for *w*. (This allows us to estimate the entropy of $\bar{s}$ without assuming that $s_{i}=\bar{s}\;\forall \;i$. The procedure is akin to introducing a “chemical potential” *w* which fixes the mean number of self-bonds.) In the thermodynamic limit the partition function is dominated by the largest term, so we minimize Eq 1 with respect to $\bar{s}$ and $\bar{t}$ at each *ϕ* to yield *f*(*ϕ*) and determine the phase diagram.

Fig 2d shows the mean-field phase diagrams. In spite of the theory’s approximations, it captures the main patterns observed in the full simulations. Specifically, sequences with larger motif blocks have larger two-phase regions and these extend to higher temperatures. (The mean-field *T*_c_ values differ from the simulations, but these could be tuned by the nonspecific-interaction parameter *χ*. Density fluctuations make it difficult to map *χ* to *J*, so we use the mean-field relation *χ* = −*V Jz*/2 for simplicity.) Rescaling by *T*_c_ and *ϕ*_c_ also causes the mean-field phase boundaries to collapse (Fig F in S1 Text). Intriguingly, the mean-field theory does not correctly place the *ℓ* = 1 sequence in the *T*_c_ hierarchy. The single-polymer density of states *g*(*s*) suggests that *ℓ* = 1 should be similar to *ℓ* = 2, but its *T*_c_ is closer to to *ℓ* = 4. We trace this discrepancy to trans-bond correlations in the dense phase: the *ℓ* = 1 sequence tends to form segments of multiple bonds rather than independent bonds (see “Dense-phase correlations” in S1 Text for details). Overall, the success of the theory demonstrates that motif sequence mainly governs phase separation through the entropy of self-interactions. We capture this dependence, as well as corrections due to dense-phase correlations, in a simple “condensation parameter” described below.

**Fig 3:**
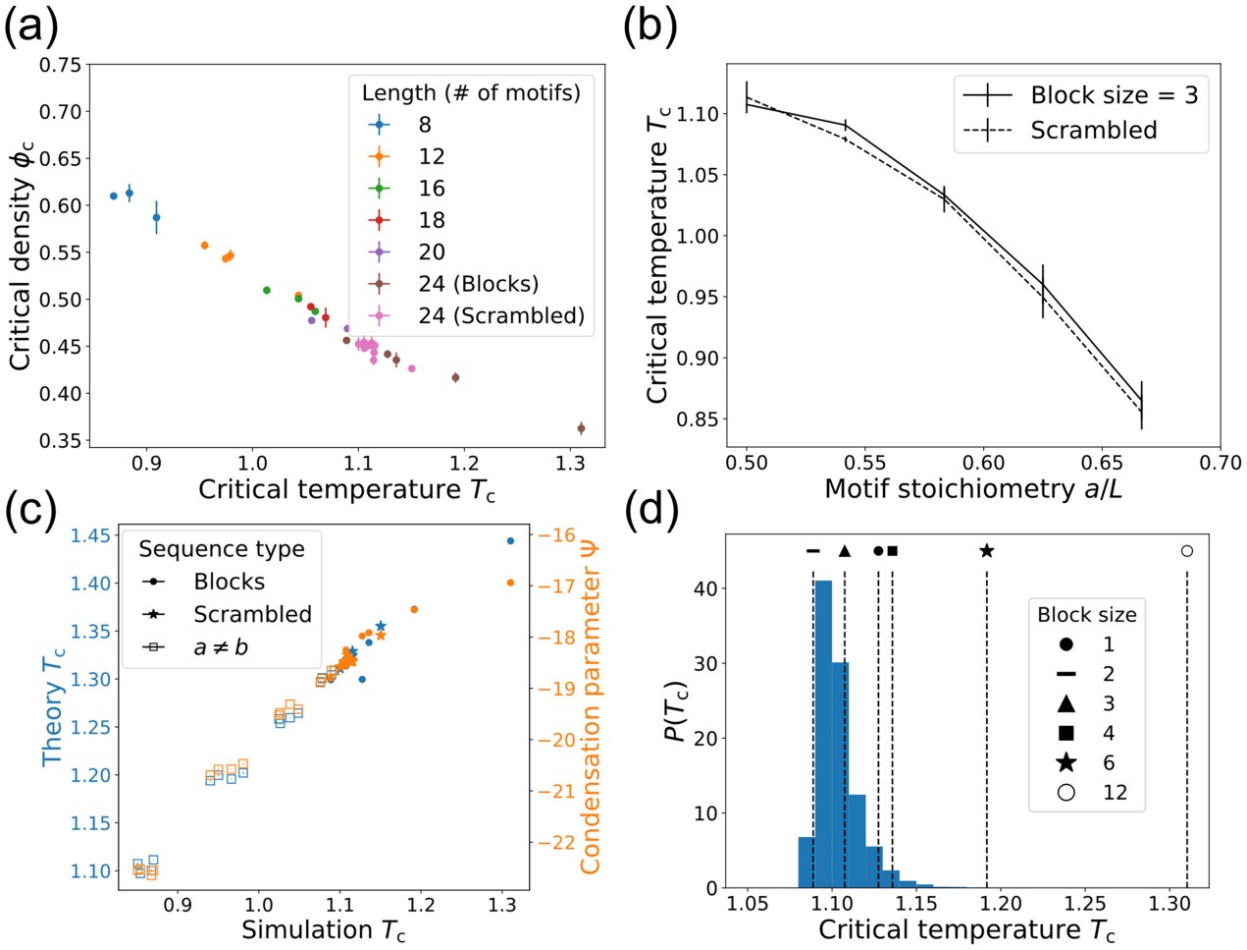
Ability to phase separate is determined by the sequence of binding motifs for polymers of different lengths, patterns, and motif stoichiometries. (a) *T*_c_ and *ϕ*_c_ for *L* = 24 polymers with scrambled sequences and block sequences of various lengths. Mean ± SD over three replicates. (Temperature uncertainties are too small to see in (a) and (c).) (b) *T*_c_ as a function of motif stoichiometry *a*/*L*. The solid curve corresponds to *ℓ* = 3 sequences where a number of B motifs are randomly mutated to A motifs, and the dashed curve shows scrambled sequences. Mean ± SD over four different sequences. (c) *T*_c_ from Monte Carlo simulations versus mean-field theory (blue) and condensation parameter (orange) for block sequences, scrambled sequences, and sequences with unequal motif stoichiometry, all *L* = 24. Mean ± SD over three replicates for simulation *T*_c_. (d) Distribution of *T*_c_ values for 20, 000 random sequences of length *L* = 24 with *a* = *b*, calculated from Ψ values and the linear *T*_c_ versus Ψ relation for block sequences. Block sequence *T*_c_ values are marked.

Do these conclusions still hold if the motifs are not arranged in regular blocks, and how do polymer length and motif stoichiometry affect phase separation? To address these questions, we located the critical points for three new types of sequences: 1) Length *L* = 24 sequences with *a* = *b* = 12 in scrambled order, 2) block sequences with *L* ≠ 24, and 3) sequences with *L* = 24 but *a* ≠ *b*. Each simulation contains only polymers of a single sequence. We find that the *T*_c_ hierarchy with respect to block size *ℓ* is preserved across sequence lengths, so block size is a robust predictor of phase separation (Fig H in S1 Text). Fig 3a shows *T*_c_ and *ϕ*_c_ for the scrambled *L* = 24 sequences and for block sequences of various lengths. *T*_c_ and *ϕ*_c_ are negatively correlated across all sequences because for low-*T*_c_ sequences, trans-bonds—and consequently, phase separation—only become favorable at higher polymer density.

The dashed curve in Fig 3b shows *T*_c_ for scrambled sequences with unequal motif stoichiometry. *T*_c_ decreases as the motif imbalance grows because the dense phase is crowded with unbonded motifs, making phase separation less favorable. How does this crowding effect interplay with the previously observed effect of *g*(*s*)? Scrambled sequences are clustered near the *ℓ* = 3 sequence in (*T*_c_, *ϕ*_c_) space (Fig G in S1 Text), so we generated sequences by starting with *ℓ* = 3 and randomly mutating B motifs into A motifs (Fig 3b, solid curve). The *ℓ* = 3 mutants follow the same pattern as the scrambled sequences, indicating that self-bond entropy and stoichiometry are nearly independent inputs to *T*_c_. This arises because motif flips have a weak effect on *g*(*s*) but a strong effect on dense phase crowding, giving cells two independent ways to control condensate formation through sequence.

The mean-field theory of Eq 1 also captures the behavior of these more general sequences, as shown in Fig 3c. The critical temperatures from theory (blue markers) correlate linearly with the simulation *T*_c_ values. (The magnitude differs, but this is tuned by the strength of nonspecific interactions.) This agreement reinforces the picture that *T*_c_ is mainly governed by the relative entropy of intra- and inter-polymer interactions. The former is captured by *g*(*s*) and the latter depends on the motif stoichiometry. To capture these effects in a single number, we propose a condensation parameter Ψ which correlates with a sequence’s ability to phase separate (see “Condensation parameter Ψ” in S1 Text for a heuristic derivation):

$$\begin{array}{c} \Psi \equiv -\;\text{log}\;(\frac{1}{{\left(r_{\text{A}}\right)}^{b}{\left(r_{\text{B}}\right)}^{a}}\sum_{s}\frac{g(s)}{{\left(4\left\langle P_{\text{corr}}\right\rangle \right)}^{s/2}}), \end{array}$$ (4)

where *r*_A_ = *a*/*L* is the fraction of motifs that are A (and likewise for *r*_B_) and 〈*P*_corr_〉 is a simple metric for trans-bond correlations (See S1 Text). A sequence with large Ψ has a high *T*_c_ because the dense phase is relatively favorable due to low self-bonding entropy, strong dense-phase correlations, or balanced motif stoichiometry. As shown in Fig 3c (orange markers), this accurately captures the phase separation hierarchy of *T*_c_, including the correlation-enhanced *T*_c_ of the *ℓ* = 1 sequence.

Are block sequences special? The space of possible sequences is much larger than can be explored via Monte Carlo simulations. However, we can use the condensation parameter to estimate *T*_c_ for any sequence without additional simulations. First, we estimate *g*(*s*) analytically and use this to approximate Ψ for new sequences. Then we use a linear fit of Ψ to the known *T*_c_ values for the block sequences to estimate the critical temperature (details in “Condensation parameter Ψ” in S1 Text). Fig 3d shows the distribution of critical temperatures calculated in this way for 20, 000 random sequences with *a* = *b* = 12. Strikingly, the distribution is sharply peaked at low *T*_c_, similar to the block sequences with *ℓ* = 2 or *ℓ* = 3. If particular condensates with high *T*_c_ are biologically beneficial, then evolution or regulation could play an important role in generating atypical sequences like *ℓ* = 12 with large two-phase regions.

**Fig 4:**
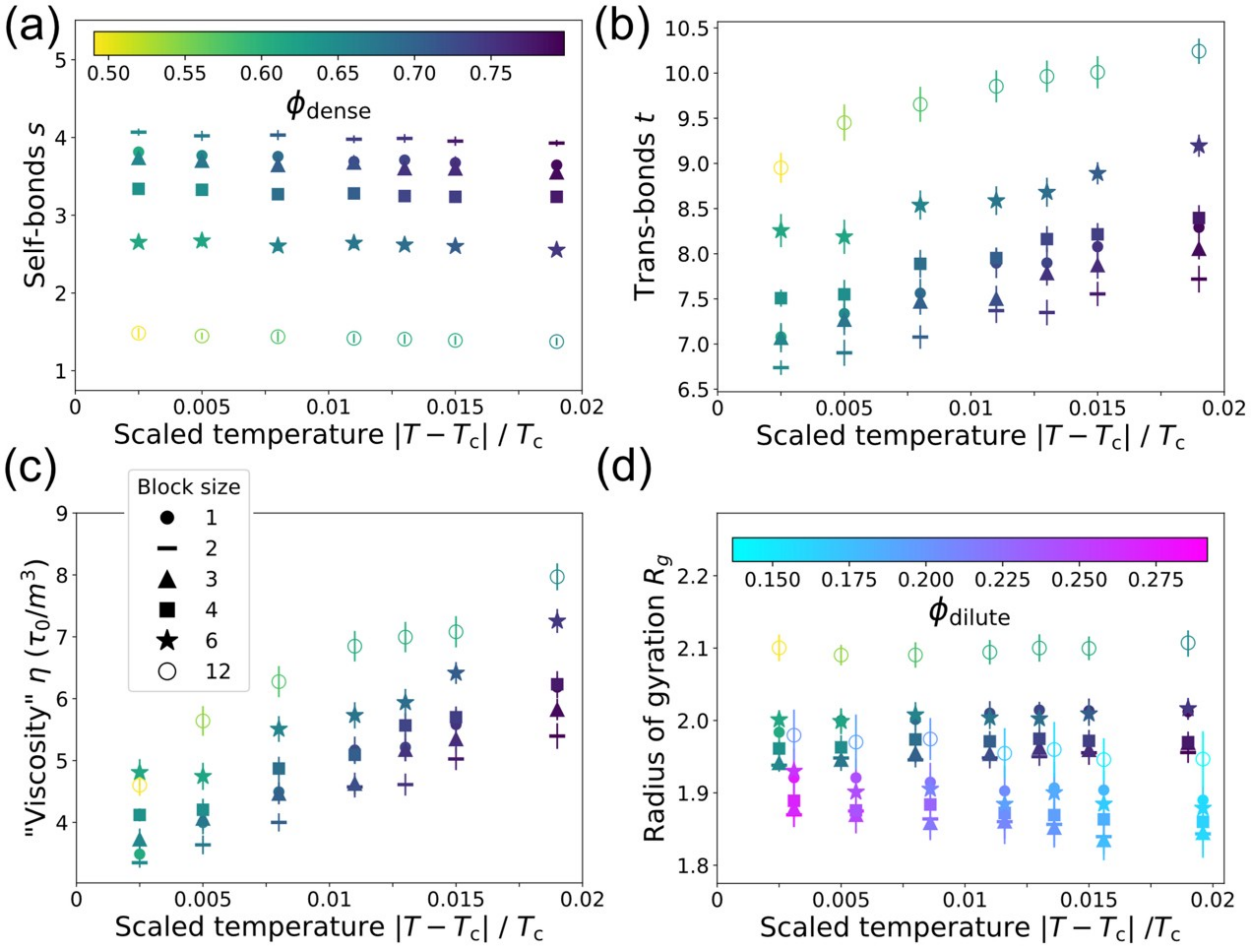
The structure of the dense phase depends on the motif sequence. (a) Number of self-bonds *s* in the dense phase as a function of reduced temperature for block sequences (symbols as in (c)). Each point shows *s* (mean ± SD) over all configurations with |*ϕ* − *ϕ*_dense_| ≤ 0.01. Color bar: droplet density. (b) Number of trans-bonds *t* (bonds with other polymers) versus temperature as in (a). (c) “Viscosity” (Eq 5) of the dense phase, shown as in (a). Symbol key applies to all panels. (d) Radius of gyration *R*_g_ of polymers in the dense phase (shown as in (a)) and in the dilute phase. Dilute-phase points show *R*_g_ (mean ± SD) over all configurations with |*ϕ* − *ϕ*_dilute_| ≤ 0.01. They share reduced temperatures with the dense phase points but are shifted for clarity. Color bar: dilute phase density.

The sequence of specific-interaction motifs influences not only the formation of droplets, but also their physical properties and biological function. Fig 4a shows the number of self-bonds in the dense phase relative to scaled temperature |*T* − *T*_c_|/*T*_c_. Density fluctuates in the GCE, so each point is averaged over configurations with *ϕ* within 0.01 of the phase boundary, and this density is indicated via the marker color (marker legend in Fig 4b). The sequence ordering of self-bonds in the dense phase matches the sequence ordering of the single-polymer *g*(*s*), indicating that sequence controls intrapolymer interactions even in the condensate. Fig 4b shows the number of trans-bonds in the dense phase, plotted as in Fig 4a. Larger blocks lead to more trans-bonds, even though the droplets are less dense. As temperature is reduced—and thus density is increased—the number of trans-bonds increases. Interestingly, even though the phase boundaries collapse to the same curve (Fig 2b), different sequences lead to droplets with very different internal structures.

These structural differences will affect the physical properties of the dense phase. The timescales of a droplet’s internal dynamics will determine whether it behaves more like a solid or a liquid. We might expect denser droplets to have slower dynamics, so the *ℓ* = 1 and *ℓ* = 2 sequences would be more solid-like. However, the extra inter-polymer bonds at large *ℓ* will slow the dynamics. To disentangle these effects, we estimate the viscosity and polymer-diffusivity by modeling the dense phase as a viscoelastic polymer melt with reversible cross-links formed by trans-bonds. Then the viscosity is expected to scale as [28]

$$\begin{array}{c} \eta ∼G\tau =(k_{\text{B}}T\frac{\phi }{m^{3}L})(\tau_{b}{\bar{t}}^{2}), \end{array}$$ (5)

where *G* is the elastic modulus, *τ* is the relaxation time of the polymer melt, and *m* is the monomer length. *τ* depends on the trans-bonds per polymer $\bar{t}$ and the bond lifetime *τ*_*b*_ = *τ*_0_ exp(*βϵ*), where *τ*_0_ is a microscopic time which we take to be sequence-independent. Fig 4c shows the dense-phase viscosity calculated using in Eq 5 the $\bar{t}$ and *ϕ*_dense_ obtained from simulation. We find that sequences with large blocks have more viscous droplets due to the strong dependence on inter-polymer bonds, in spite of their substantially lower droplet density. (See the S1 Text for off-lattice molecular-dynamics simulations that directly verify this conclusion.) By the same arguments leading to Eq 5, diffusivity scales as $1/\bar{t}$, so polymers with large blocks will also diffuse more slowly within droplets (Fig I in S1 Text). Thus trans-bonds are the main repository of elastic “memory” in the droplet.

The motif sequence also affects the polymer radius of gyration in both phases (Fig 4d). In the dense phase, polymers with large blocks adopt expanded conformations which allow them to form more trans-bonds. Polymers of all sequences are more compact in the dilute phase, where there are fewer trans-bonds and nonspecific interactions with neighbors. Thus self-bonds cause polymers to contract, while trans-bonds cause them to expand.

## 3 Discussion

In summary, we developed a simple lattice-polymer model to study how the sequence of specific-interaction motifs affects phase separation. We found that motif sequence determines the size of the two-phase region by setting the relative entropy of intra- versus inter-molecular bonds. In particular, large blocks of a single motif disfavor self-bonds and thus favor phase separation. This is consistent with recent experimental [18] and theoretical [12–14] studies on coacervation (phase separation driven by electrostatics) where small charge-blocks lead to screening of the attractive forces driving aggregation. However, electrostatic interactions (generic, longer-range, promiscuous) are qualitatively very different from the interactions in our model (specific, local, saturating). This points to a different underlying mechanism: in the former, sequence primarily influences the electrostatic energy of the dense phase, but in the latter, sequence controls the conformational entropy of the dilute phase. Thus specific interactions provide a distinct physical paradigm for the control of intracellular phase separation. While our dilute phase concentrations are large relative to experimental values due to weak nonspecific interactions and the discrete lattice, we expect these sequence-dependent patterns to be quite general. If anything, the self-bond entropy will be even more important at low *ϕ*_dilute_. The saturating nature of bonds in our model also explains why we do not observe the spatially-structured aggregates (e.g. micelles and membranes) reported for sequences of hydrophobic motifs [20]. In these structured aggregates, hydrophobic motifs can interact with multiple neighbors, which compensates the loss of entropy—by contrast, specific-interaction motifs can only interact with one neighbor at a time.

These results shed light on several recent experiments. Schuster et al. showed that phase separation by the disordered region of LAF-1, a commonly studied IDP found in the P granules of *Caenorhabditis elegans* embryos, depends on the sequence of tyrosines and arginines [29]. Wild-type LAF-1 has tyrosines and arginines distributed evenly throughout the sequence, and Schuster et al. showed that LAF-1 mutants with large blocks of tyrosines and arginines are much better at phase separating. They attributed this to charged interactions, but mutating the arginines to another cation (lysine) disrupted phase separation, so it is likely that specific interactions between tyrosine and arginine are also important. Thus, their results are consistent with our prediction that large blocks of specific-interaction motifs promote phase separation due to the low entropy of self-interactions. We have focused on proteins, but similar physical principles may also be relevant in RNA systems, where secondary structure depends on specific self-interactions. Secondary structure can control whether a transcript remains in the dilute phase or enters a condensate [30], suggesting that the entropy of self-interactions may influence transcript partitioning. The entropy of self-interactions could also drive RNA aggregation in disease, where transcripts with nucleotide repeats phase separate more readily than scrambled sequences [26]. It will be interesting to ask how these observations relate to the robust phase separation of large-block sequences in the present work. Moreover, models with explicit solvent molecules and counterions show that the entropy of solvation has a strong sequence dependence [19], and it will be worthwhile to consider how this effect modulates the conformational entropy studied here.

We then analyzed how sequence influences condensates’ physical properties such as viscosity and diffusivity. We found that motif sequence strongly affects both droplet density and inter-polymer connectivity, and, in particular, that sequences with large blocks form more viscous droplets with slower internal diffusion because they form more trans-bonds. This generalizes the recent finding that higher binding-motif valency slows down particle exchange [31]. In both cases, the underlying cause of slow dynamics is the formation of trans-bonds, which in our case is influenced via sequence rather than valency. Because the viscoelastic properties of our system depend strongly on self-binding entropy, the density and viscosity of the dense phase are not necessarily correlated. This is intriguing in light of recent experiments showing that changes in motif identity drive density and viscosity in the same direction [32, 33], because it suggests that the specific sequence of motifs could provide an orthogonal mechanism of control that decouples density and viscosity. For our simulated polymers, all sequences expand in the dense phase to form more trans-bonds, and small-block sequences are the most compact. This contrasts with results for single polyampholyte chains, where sequences with large charge blocks are more compact [34, 35]. The difference arises because our system includes many polymers interacting with each other and because hairpins are less favored by specific bonds than by longer-range electrostatic interactions.

Taken together, these results suggest that motif sequence provides cells with a means to tune the formation and properties of intracellular condensates. For example, motif stoichiometry could be an active regulatory target—a cell could dissolve droplets by removing just a few binding motifs per polymer through post-translational modifications. The negative correlation between *T*_c_ and *ϕ*_c_ provides another regulatory knob: if a particular condensate density is required at fixed temperature, this can be achieved by either tuning the binding strength or modifying the sequence. However, the physics also implies biological constraints: the same trans-bonds that drive condensation for high-*T*_c_ sequences also lead to high viscosity, which may not be functionally favorable. Such trade-offs are informative in light of recent proposals that droplet function requires a delicate balance between dynamics and structural stability [36]. Looking beyond viscosity, for some prion-forming proteins, the liquid phase is metastable with respect to a solid, aggregate phase [37], and the role of sequence in governing that transition is an exciting avenue for future research. Sequence also influences the network structure of the dense phase, where most sequences form few correlated bonds but a small subset (such as *ℓ* = 1 and *ℓ* = 12) form longer aligned segments. It has recently been shown that such aligned “zippers” can tune functional properties such as client recruitment [38], providing another link between sequence and function.

In spite of the simplicity of our model, it makes several concrete predictions relevant for both natural and engineered systems. In particular, we predict that the condensates of sequences with large blocks of specific-interaction motifs will be less dense and more viscous, with higher critical temperatures. This can be tested directly with IDPs via mutation experiments or with synthetic biopolymers whose interaction motifs are arranged in blocks of different sizes (e.g. using the SIM-SUMO or SH3-PRM systems). Of course, different mechanisms could lead to similar macroscopic effects. How can we test whether sequence acts via the entropy of self-interactions or the energy of trans-interactions? Recently, Isothermal Titration Calorimetry (ITC) was used to measure the relative contributions of electrostatic energy and solvation entropy upon formation of a complex coacervate [19]. ITC could be used in a similar way with sequences of specific-interaction motifs. Specifically, experimenters could titrate dilute polymers into a reaction cell containing a condensate and measure the energy input necessary to maintain the same temperature as a reference cell. Analyzing the slope and integral of the energy curve would reveal the change in energy and entropy as polymers enter the dense phase. Our model predicts that all sequences will undergo a large decrease in energy as trans-bonds form, but each sequence will have a distinct entropy change due to the entropy of self-bonds. Thus, ITC is a promising technique to test our proposed role for sequence in determining the entropy of self-interactions.

We have used a simple model of biological condensates to show how the sequence of specific-interaction motifs affects phase separation, thus linking the microscopic details of molecular components to the emergent properties relevant for biological function. What lessons are likely to generalize beyond the details of the model? When nonspecific interactions dominate, forming a dense droplet has a large energetic payoff. When interactions are specific and saturating, however, the energy change is limited and the conformational entropy is expected to play a bigger role. For example, in two-component systems the conformational entropy of small oligimers can stabilize the dilute phase [25, 39], or the conformational entropy of gelation can stabilize a dense phase [40], depending on the molecular architecture. Here, we have shown that the conformational entropy of self-interactions can play a similar role, and we use the density of states *g*(*s*) to connect sequence and entropy. Understanding the general role of the entropy of self-interactions will prove useful if it allows us to gain insight into biomolecular phase separation by simply analyzing the properties of single molecules or small oligomers rather than necessarily tackling the full many-body problem. Many open questions remain, however, and we hope our work encourages further research across a range of theoretical and experimental systems.

## 4 Methods and materials

**Fig 5:**
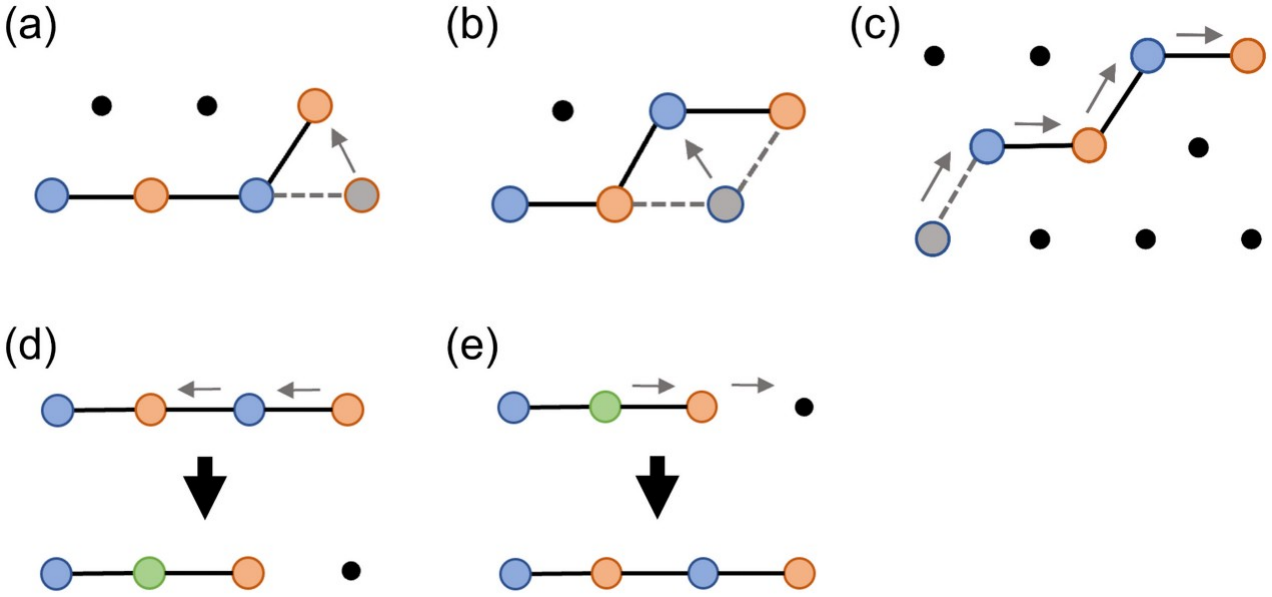
The polymer moves used to update Monte Carlo simulations at each step. We also allow translation of connected clusters of polymers and insertion/deletion of polymers. (a) End move. (b) Corner move. (c) Reptation. (d) Contraction. (e) Expansion.

We performed Monte Carlo simulations in the Grand Canonical Ensemble on a 30 × 30 × 30 FCC lattice, corresponding to a volume of *V* = 30^3^ lattice sites, with periodic boundary conditions. When “A” and “B” monomers occupy the same site, they form a bond with energy *ϵ*. Other overlaps are forbidden. When two monomers of any type occupy adjacent lattice sites, they have an attractive nonspecific interaction energy *J*. Thus each lattice site *i* has a bond occupancy *q*_*i*_ ∈ [0, 1] and a motif occupancy *r*_*i*_ ∈ [0, 1, 2]. The Hamiltonian for our system is therefore

$$\begin{array}{c} H=-\epsilon \sum_{i}q_{i}-J\sum_{\left\{i,j\right\}}r_{i}r_{j}, \end{array}$$ (6)

where the brackets indicate summation over adjacent lattice sites. Each simulation has fixed control variables *β* = 1/*k*_B_*T* and polymer chemical potential *μ*. All simulations use *ϵ* = 1 and *J* = 0.05*βϵ*, so nonspecific interactions are weak relative to specific interactions. We initialize the simulation with *N* = 100 polymers. Each polymer is initialized as a randomly-placed straight line of monomers to avoid knots. If placing a monomer would result in a forbidden overlap, then a random new direction is chosen for the rest of the polymer. We use simulated annealing to cool the system to the final temperature, and after reaching that temperature to ensure the system has thermalized we only use data from the last 80% of steps. The total number of Monte Carlo steps varies, but is around 4.5 ⋅ 10^8^ for critical point simulations and 3 ⋅ 10^8^ for binodal simulations. In each Monte Carlo step, we update the system configuration by proposing a move from the move-set defined in Fig 5. Fig 5a, 5b and 5c show standard polymer moves. We include contraction and expansion moves (Fig 5d and 5e) which allow contiguous motifs to form and break bonds. The FCC lattice has coordination number *z* = 12, so there are 12 states that can transition into any one contracted state. Thus it is necessary to propose expansions at 12 times the rate of contractions to satisfy detailed balance. We also allow clusters of polymers connected by A-B overlap to translate by one site so long as no overlap bonds are formed or broken.

To include insertions and deletions of polymers, we assume the existence of a reservoir of polymers of chemical potential *μ*, which we can adjust. Because inserting a polymer tends to increase the configurational entropy of the system, we adopt the common convention of shifting *μ* by the entropy of an ideal polymer: *μ* ≡ *μ*_0_ + ln(*z* + 1)^*L*−1^, where the “+1” in *z* + 1 comes from allowing the “walk” to remain on the same site and form a contiguous bond (see Fig 5d and 5e). We then remove the shift with a prefactor in the acceptance probabilities (Eq 12). This convention allows us to simulate the dilute phase without setting *μ* to a large negative value.

In our Monte Carlo move set, we allow for the deletion of any polymer, and require that insertion moves satisfy detailed balance with respect to deletions. This still allows for considerable freedom in the insertion algorithm. Naively, we might insert polymers as random walks, but for a dense system most such random walks will be disallowed because of forbidden overlaps. For efficiency, we therefore implemented a form of Configurational-Bias Monte Carlo (CBMC) [41]. Specifically, we insert the head of a polymer at a randomly chosen site, and then perform a biased walk along an allowed path, keeping track of the number of available choices at each step to generate a “Rosenbluth weight” *R*:

$$\begin{array}{c} R=\prod_{k=1}^{L-1}W_{k}, \end{array}$$ (7)

where *W*_*k*_ is the number of allowed sites for monomer *k* + 1 starting from the position of monomer *k*. The probability of this insertion move is therefore

$$\begin{array}{c} P_{\text{insert}}=\frac{1}{V}\frac{1}{R}. \end{array}$$ (8)

The CBMC algorithm satisfies detailed balance so long as the net flow of probability between any two configurations *x*_1_ and *x*_2_ is zero. In words, this imposes the condition

$$\begin{array}{c} \begin{array}{cc} & P(\text{being}\;\text{in}\;x_{1})\times P(\text{proposing}\;x_{2})\times P(\text{accepting}\;x_{1}\to x_{2})=\\ & P(\text{being}\;\text{in}\;x_{2})\times P(\text{proposing}\;x_{1})\times P(\text{accepting}\;x_{2}\to x_{1}). \end{array} \end{array}$$ (9)

In our system, if configuration *x*_1_ has polymer number *N* and energy *E*_*N*_ and *x*_2_ has polymer number *N* + 1 and energy *E*_*N*+1_, Eq 9 becomes

$$\begin{array}{c} P\left(E_{N},N\right)\times P_{\text{insert}}\times P_{\text{acc}}\left(\Delta N=+1\right)=P\left(E_{N+1},N+1\right)\times P_{\text{delete}}\times P_{\text{acc}}\left(\Delta N=-1\right), \end{array}$$ (10)

where *P*(*E*, *N*) = exp(−*βE* + *βμN*)/*Z* is the equilibrium probability of the state. CBMC leads to the *P*_insert_ in Eq 8. *P*_delete_ = 1/(*N* + 1), because polymers are chosen randomly for deletion. This leads to the following condition on the acceptance probabilities:

$$\begin{array}{c} P_{\text{acc}}\left(\Delta N=+1\right)=\frac{VR}{N+1}\;\text{exp}\;(-\beta (E_{N+1}-E_{N}-\mu ))\;P_{\text{acc}}\left(\Delta N=-1\right). \end{array}$$ (11)

The acceptance probabilities given below in Eq 12 satisfy this condition and also incorporate the multicanonical sampling described next.

**Fig 6:**
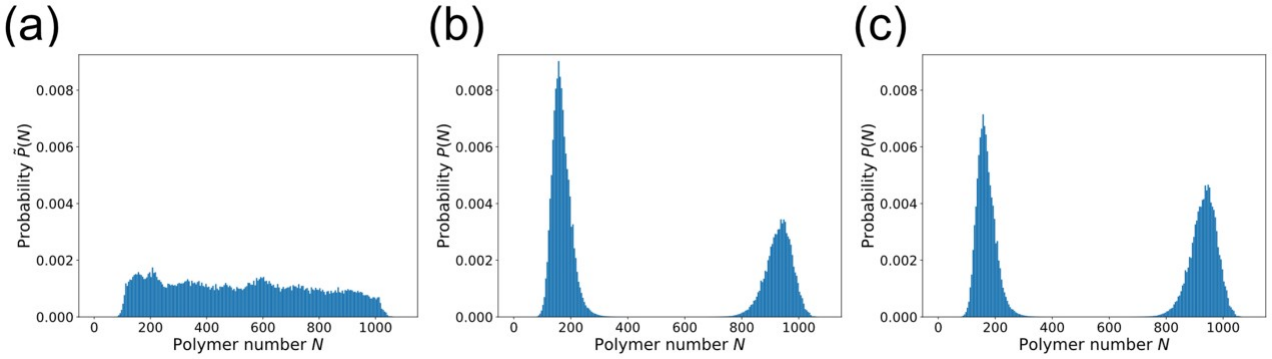
Multicanonical sampling makes it possible to determine the phase boundary at temperatures substantially below *T*_c_. (a) The polymer number distribution $\tilde{P}\left(N\right)$ produced in a multicanonical simulation with $\tilde{H}=H+h\left(N\right)$. Block sequence with *ℓ* = 2, *βϵ* ≈ 0.94, *J* = 0.05*ϵ*. (b) The true distribution *P*(*N*), obtained by reweighting $\tilde{P}\left(N\right)$ from (a) to remove *h*(*N*). (c) The distribution at the phase boundary, obtained by reweighting (b) to the chemical potential μ* at which both peaks have equal weight.

We determine the phase diagram using histogram reweighting [27] of *P*(*N*, *E*), where *N* is the polymer number and *E* is the total system energy. This allows us to extrapolate a histogram *P*(*N*, *E*) obtained at *β*_0_, *μ*_0_ to $\tilde{P}\left(N,E\right)$ at nearby *β*_1_, *μ*_1_. First we determine the approximate location of the critical point by locating the parameters where *P*(*N*) resembles two overlapping Gaussians (indicative of rapid transitions between phases and low surface tension), then run a sufficiently long simulation to obtain a converged *P*(*N*, *E*). We determine the exact location of the critical point by finding the *β*_c_, *μ*_c_ where $\tilde{P}\left(N,E\right)$ matches the universal distribution known for the 3D Ising model [42]. (Because polymer models lack the symmetry of the Ising model, we also must fit a “mixing parameter” *x* which determines the order parameter *N* − *xE* [43].) In principle, we could find the binodal at temperature *T* < *T*_c_ (*β* > *β*_c_) by determining *P*_*β*_(*N*, *E*), then reweighting the histogram to the μ* at which *P*_*β*_(*N*) has two peaks with equal weight. The phase boundaries *ϕ*_dilute_ and *ϕ*_dense_ would then be the means of these peaks, which we could find by fitting *P*_*β*_(*N*) to a Gaussian mixture model. However, determining the relative equilibrium weights of the two phases requires observing many transition events, which are very rare at temperatures substantially below *T*_c_. To circumvent this difficulty, we use multicanonical sampling [43]: Once we have $P_{\beta_{\text{c}}}\left(N,E\right)$ at the critical point, we use reweighting to estimate ${\tilde{P}}_{\beta_{1}}\left(N,E\right)$ at a slightly lower temperature *β*_1_. When we perform the new simulation at *β*_1_, we use a modified Hamiltonian $\tilde{H}=H+h\left(N\right)$, where $h\left(N\right)=\frac{1}{\beta_{1}}\;\text{log}\;{\tilde{P}}_{\beta_{1}}\left(N\right)$. (Note that *h*(*N*) is only defined over the range of *N* between the two peaks.) This yields ${\tilde{P}}_{\beta_{1}}\left(N\right)$, which is unimodal and flat-topped with respect to *N* rather than bimodal, and thus allows rapid sampling of the full range of relevant values of *N*. Fig 6a shows an example distribution $\tilde{P}\left(N\right)$. Finally, we use reweighting to remove *h*(*N*) and study the true histogram $P_{\beta_{1}}\left(N,E\right)$, as in Fig 6b. We apply this procedure iteratively to obtain the phase boundary at lower and lower temperatures. Combining multicanonical sampling with Configurational-Bias Monte Carlo, our acceptance probabilities become

$$\begin{array}{c} P_{\text{acc}}=\{\begin{array}{cc} \text{min}\{1,\;\text{exp}\;(-\beta \Delta H)\} & \Delta N=0\\ \text{min}\{1,\frac{V}{N+1}\frac{R}{{\left(z+1\right)}^{L-1}}\;\text{exp}\;(-\beta (\Delta H-\mu \Delta N)-\beta (h(N+1)-h(N)))\} & \Delta N=+1\\ \text{min}\{1,\frac{N}{V}\frac{{\left(z+1\right)}^{L-1}}{R}\;\text{exp}\;(-\beta (\Delta H-\mu \Delta N)-\beta (h(N-1)-h(N))\} & \Delta N=-1 \end{array} \end{array}$$ (12)

*Single-polymer properties*. The density of states *g*(*s*) is the number of configurations of an isolated polymer with *s* self-bonds. We extract *g*(*s*) by performing Monte Carlo simulations of the polymer over a range of *β* values. The distributions are then combined using the multihistogram method, and inverted to determine the density of states [44].

## Supporting information

S1 Text Supplementary simulations, analysis, and derivations. (PDF)
