# Supplementary material for: Motif-pattern dependence of biomolecular phase separation driven by specific interactions
Source: PLoS Comput Biol. 2021 Dec 29;17(12):e1009748. doi: 10.1371/journal.pcbi.1009748 (PMC8751999; doi:10.1371/journal.pcbi.1009748)
Supplement: S1 Text — (PDF) [file pcbi.1009748.s001.pdf]

# Supporting Information for: Motif-pattern dependence of biomolecular phase separation driven by specific interactions

Benjamin G. Weiner [1], Andrew G. T. Pyo [1], Yigal Meir [1,2], Ned S. Wingreen [3,4*]

**1** Department of Physics, Princeton University, Princeton, New Jersey, USA
**2** Department of Physics, Ben Gurion University of the Negev, Beer-Sheva, Israel
**3** Department of Molecular Biology, Princeton University, Princeton, New Jersey, USA
**4** Lewis-Sigler Institute for Integrative Genomics, Princeton University, Princeton, New Jersey, USA

## S1A Mean-field theory

We aim to find the partition function $Z$ for a system with $N$ identical, interacting polymers on a lattice with $V$ sites. Each polymer has $a$ A motifs, $b$ B motifs, and length $L = a + b$. We label the state of polymer $i$ by the number of self-bonds $s_i$ and trans-bonds $t_i$. Then the total number of self-bonds is $S \equiv \sum_i s_i$, and the total number of trans-bonds is $T \equiv \frac{1}{2}\sum_i t_i$. In our approach, each polymer forms self-bonds according to its own full degrees of freedom encoded in the density of states $g(s)$. However, we approximate the inter-polymer interactions within a mean-field approach. The full partition function for our system is then given by

$$Z = \sum_{\xi,T} n(\xi,T) e^{\beta\epsilon(\xi+T)+\beta\chi\phi^2} \sum_{\{S=\xi\}} \left( \prod_i^N g(s_i) \right),$$

where $n(\xi, T)$ is the combinatorial term for counting states with $T$ A-B overlap bonds (given $\xi$ total self-bonds) and the second sum is over all configurations where $S = \xi$. The parameter $\chi$ quantifies the strength of two-body nonspecific interactions, e.g. as appears in Flory-Huggins theory. We make the approximation that in the thermodynamic limit, $Z$ is dominated by the largest term:

$$Z \approx \max_{\xi,T} \left[ n(\xi,T) e^{\beta\epsilon(\xi+T)+\beta\chi\phi^2} \sum_{\{S=\xi\}} \left( \prod_i^{N_P} g(s_i) \right) \right], \tag{S1A}$$

$$\beta F \approx \min_{\xi,T} \left[ -\log\left( n(\xi,T) e^{\beta\epsilon(\xi+T)+\beta\chi\phi^2} \right) - \log G(\xi) \right], \tag{S1B}$$

where $G(\xi)$ is the entropy associated with forming $S = \xi$ self-bonds.

First we calculate $n(\xi, T) = n_{\text{steric}} \times n_{\text{trans}}$. $n_{\text{steric}}$ is the number of allowed ways to place the polymers on the lattice and $n_{\text{trans}}$ is the number of ways to form $T$ trans-bonds. To find $n_{\text{steric}}$, we ignore chain connectivity and simply count the number of ways of choosing $N\langle l \rangle$ sites on a lattice with $V$ sites, where

$$\langle l \rangle = L - \bar{s} - \bar{t}/2 \tag{S1C}$$

is the mean number of sites occupied by a polymer. We account for excluded volume using a semi-dilute approximation that the probability of placing monomer $k$ successfully is the fraction of empty sites remaining:

$$n_{\text{steric}} = \binom{V}{N} \prod_{k=N}^{N(\langle l \rangle - 1)} \frac{V-k}{V}, \tag{S1D}$$

where $\binom{V}{N}$ counts the center-of-mass, or equivalently "polymer head," degrees of freedom. We find $n_{\text{trans}}$ by assuming that each protein sees the others as a mean-field cloud of motifs with which it can form A-B

overlap bonds depending on the overall motif density. Then

$$n_{\text{trans}} = \binom{Na - S}{T}\binom{Nb - S}{T} T! \left(\frac{1}{V}\right)^T, \tag{S1E}$$

where the first two terms count the number of ways to choose $T$ A motifs and $T$ B motifs, given that $S$ of each are already in self-bonds. $T!$ is the number of ways to pair the chosen motifs, and the final term is the mean-field probability that two motifs are close enough to form a bond. (This is simply an extension of Semenov and Rubinstein's sticker model to two sticker types on a lattice [1].)

Now we calculate $F_G(\xi) \equiv -\log G(\xi)$, the entropy of having exactly $S = \xi$ self-bonds. The difficulty arises from the restricted sum: we only want to count states with the correct total number of self-bonds. However, we can relax this restriction and require instead that $\langle S \rangle = \xi$. Formally, this is equivalent to working in a "Grand Canonical Ensemble" for self-bonds, where a reservoir imposes a chemical potential $w$. In the thermodynamic limit, fluctuations vanish and all ensembles yield equivalent macrostates. Thus we can calculate $\beta\Omega = -\log Z_{\text{gc}}$ (where $\Omega$ is the grand potential and $Z_{\text{gc}}$ the grand canonical partition function), and use the Legendre transform $F_G(\xi) = \Omega + w\xi/\beta$.

Calculating $Z_{\text{gc}}$ is relatively straightforward:

$$\begin{aligned} Z_{\text{gc}} &= \sum_S e^{wS} G(S), \\ &= \left(\sum_{s_i} g(s_i) e^{ws_i}\right)^N. \end{aligned} \tag{S1F}$$

Then $w = w(\xi)$ is fixed by requiring that $\langle S \rangle = \xi$. Recall that $\overline{s} = \xi/N$, so

$$\begin{aligned} \frac{\beta F_G}{V} &= -\frac{N}{V} \log\left(\sum_{s_i} g(s_i) e^{ws_i}\right) + w\frac{\xi}{V}, \\ &= -\frac{\phi}{L} \log\left(\sum_{s_i} g(s_i) e^{ws_i}\right) + \frac{\phi}{L} w\overline{s}, \end{aligned} \tag{S1G}$$

where $\phi$ is the monomer density $NL/V$. Combining this with Eqs. S1D and S1E, we obtain the full free-energy density:

$$f \equiv \frac{\beta F}{V} = f_{\text{steric}}(\overline{s}, \overline{t}) + f_{\text{trans}}(\overline{s}, \overline{t}) + \beta\chi\phi^2 - \frac{\phi}{L}\left(\log \sum_s g(s) e^{ws}\right) + \frac{\phi}{L} w\overline{s} - \frac{\phi}{L}\beta\epsilon\left(\overline{s} + \frac{\overline{t}}{2}\right), \tag{S1H}$$

where

$$f_{\text{steric}} \equiv \frac{\phi}{L}\log\frac{\phi}{L} + \left(1 - \phi\frac{\langle l \rangle}{L}\right)\log\left(1 - \phi\frac{\langle l \rangle}{L}\right) + \frac{\phi}{L}\left(\langle l \rangle - 1\right) \tag{S1I}$$

and

$$\begin{aligned} f_{\text{trans}} &\equiv \frac{\phi}{L}\left(y(a) + y(b) + \frac{\overline{t}}{2}\log\frac{\overline{t}}{2} + \frac{\overline{t}}{2}(1 - \log\frac{\phi}{L})\right), \\ y(x) &\equiv (x - \overline{s} - \overline{t}/2)\log(x - \overline{s} - \overline{t}/2) - (x - \overline{s})\log(x - \overline{s}). \end{aligned} \tag{S1J}$$

At every $\phi$, we evaluate Eq. S1H with the average bond values $(\overline{s}^*(\phi), \overline{t}^*(\phi))$ which minimize $f$ and the $w$ which fixes $\langle s \rangle = \overline{s}$. ($\overline{s}^*(\phi)$ and $\overline{t}^*(\phi)$ are found by numerically solving $\frac{\partial f}{\partial \overline{s}} = \frac{\partial f}{\partial \overline{t}} = 0$ in Mathematica.) This yields $f(\phi)$ which we use to calculate the binodal and spinodal curves.

Regarding the nonspecific interaction parameter $\chi$, density fluctuations make it difficult to map the simulation $J$ to $\chi$, so we simply use the mean-field relation $\chi = -VJz/2$, where $z$ is the lattice coordination number. This yields theoretical $T_c$ values which differ numerically from the simulations but accurately reproduce the sequence hierarchy.

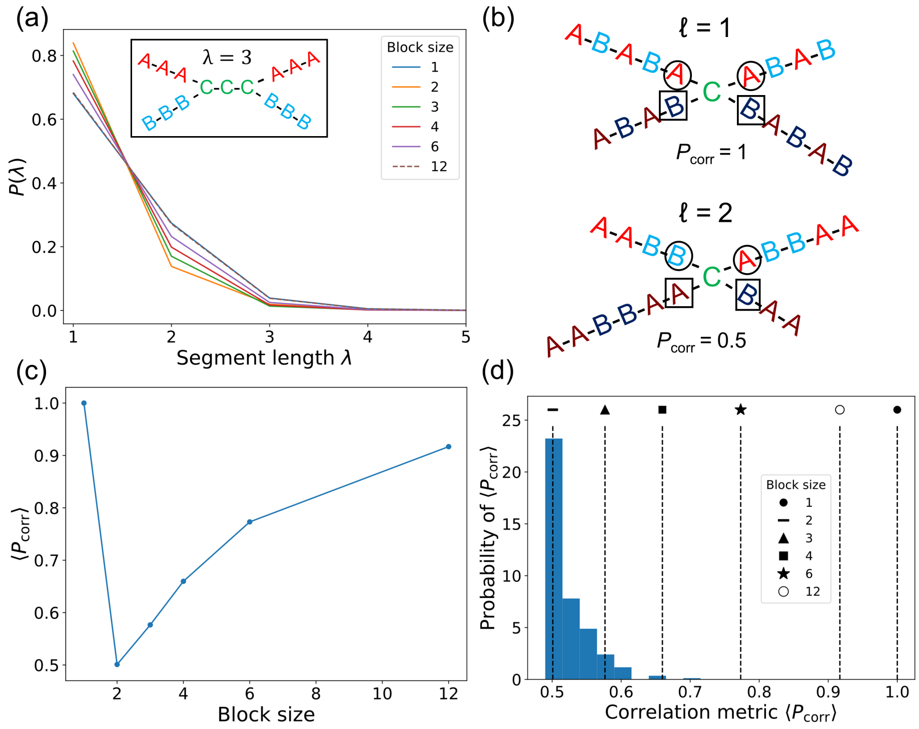


Figure A: The $\ell = 1$ polymer has correlated trans-bonds in the dense phase. (a) Probability that a trans-bond is in a segment of length $\lambda$, meaning it has $\lambda$ bonds with the same partner, and all $\lambda$ monomers are contiguous on both polymers. Data from snapshots of an NVT simulation with $\phi = 0.3$, $\beta\epsilon = 1.25$, $J = 0.05\epsilon$. *Inset:* A trans-bond segment of $\lambda = 3$, between one polymer with $(a, b) = (9, 0)$ and another polymer with $(a, b) = (0, 9)$. (b) Example $P_{\text{corr}}$ for bonds between $\ell = 1$ (*top*) and $\ell = 2$ (*bottom*) polymers. Motifs from polymer 1 and 2 are distinguished by lighter and darker shades, respectively. Bond-adjacent monomers are marked by circles for polymer 1 and squares for polymer 2. The pictured bond's $P_{\text{corr}}$ is the fraction of square-circle pairs that are A-B. (c) Trans-bond correlation probability $\langle P_{\text{corr}} \rangle$ for block sequences, where the brackets denote averaging over initial bonds. (d) Distribution of $\langle P_{\text{corr}} \rangle$ for 20,000 scrambled sequences with $a = b = 12$. Values for the block sequences are marked.

## S1B Dense-phase correlations

From simulations, the $\ell = 1$ sequence has a $T_c$ between that of $\ell = 3$ and $\ell = 4$, whereas the mean-field theory predicts that $\ell = 1$ would have a $T_c$ very close to that for $\ell = 2$. Why is the $\ell = 1$ sequence better at phase separating than the mean-field theory predicts? In the theory, sequence only appears in $g(s)$, the density of states for self-bonds. We thus assume that sequence does not directly affect inter-polymer interactions and that trans-bonds are uncorrelated. However, this assumption neglects the fact that a bond is between two polymers. We can quantify this correlation by looking at trans-bond "segments." Trans-bonds are considered to be in a segment of length $\lambda$ if two polymers have $\lambda$ trans-bonds, and all involved monomers are contiguous on both polymers (Fig A(a) *Inset*). Essentially, trans-bond segments form when two polymers are lying on top of each other. Fig A(a) shows the probability that each trans-bond is in a segment of length $\lambda$ in an NVT simulation with $\phi = 0.3$. For all sequences, the most probable segment length is 1. However, $\ell = 1$ and $\ell = 12$ both have relatively high probabilities of forming longer segments (these two curves overlap). As a result of these correlations, the dense phase is more favorable for these sequences than is predicted by the theory, and this leads to their higher $T_c$ values.

We can quantify a sequence's tendency to form correlated segment bonds by defining a correlation probability $P_{\text{corr}}$. Consider two polymers which form a bond between monomers $i$ and $j$. Now pair up neighboring monomers: the four unique possibilities are $(i-1, j-1)$, $(i-1, j+1)$, $(i+1, j-1)$, and $(i+1, j+1)$. $P_{\text{corr}}$

is the probability that these monomers will form a valid A-B bond instead of an invalid overlap. Fig A(b) shows examples for $\ell = 1$ and $\ell = 2$ sequences. Every possible initial bond $(i, j)$ has its own $P_{\rm corr}$, and so we average this $P_{\rm corr}$ over all possible bonds. This yields $\langle P_{\rm corr}\rangle$, a sequence-specific metric for trans-bond correlations. Fig A(c) shows $\langle P_{\rm corr}\rangle$ for the block sequences, and we observe that it is monotonic in block size *except* for $\ell = 1$, which has a $\langle P_{\rm corr}\rangle$ similar to $\ell = 12$. This explains why these two sequences have similar segment probabilities in Fig A(a), and why $\ell = 1$ is better at phase separating than expected from $g(s)$ alone. In Condensation parameter Ψ below, we incorporate $\langle P_{\rm corr}\rangle$ into a "condensation parameter" that successfully predicts the $T_{\rm c}$ hierarchy observed in simulation. Fig A(d) shows the distribution of $\langle P_{\rm corr}\rangle$ for 20,000 random sequences with $a = b = 12$. The distribution is strongly peaked at low values, comparable to the $\ell = 2$ sequence. This suggests that the $\ell = 1$ and $\ell = 12$ block sequences are atypical in their tendency to form correlated trans-bonds, so the mean-field theory that neglects these correlations should perform well for generic sequences.

## S1C Condensation parameter Ψ

Although our mean-field theory does a good job explaining sequence-driven patterns in $T_{\rm c}$, it would be convenient to have an order parameter that is simpler to compute but that retains some of the same predictive power. According to our results, such a metric should take into account the density of states $g(s)$, the motif stoichiometry $a, b$, and the correlation metric $\langle P_{\rm corr}\rangle$. Thus we propose as a metric the condation parameter $\Psi$:

$$\Psi \equiv -\log\left(\frac{1}{(r_{\rm A})^b (r_{\rm B})^a}\sum_s \frac{g(s)}{(4\langle P_{\rm corr}\rangle)^{s/2}}\right), \tag{S1K}$$

where the motif ratios are given by $r_{\rm A} = a/L$ and $r_{\rm B} = b/L$. The role of $g(s)$ is intuitive: the easier it is to form self-bonds, the less a polymer will tend to condense. The factor $r_{\rm A}^b r_{\rm B}^a$ characterizes the probability of placing $a$ A motifs and $b$ B motifs in the dense phase without disallowed overlap. (The mean-field motif placement probability depends on the density $\phi$, but this effect is not sequence-dependent.) Finally, we normalize $g(s)$ by the tendency to form correlated trans-bonds in the dense phase. This tendency enhances the favorability of the dense phase, and we quantify it with $\langle P_{\rm corr}\rangle$. The factor of 1/2 in $s/2$ is due to the fact that two trans-bonds/polymer are required to lower the energy by $\epsilon$/polymer, and the factor of 4 is the number of pairs of bond-adjacent monomers (Fig A(b)). Although this metric is only heuristic, it successfully captures the $T_{\rm c}$ patterns without multi-polymer simulations (Fig. 3(c)).

One limitation of the condensation parameter is that it still requires knowledge of $g(s)$ for each sequence. Is it possible to characterize the tendency of a sequence to phase separate without any simulations? In Fig. 3(d) of the main text, we replace $\sum_s g(s)$ with a theoretical calculation of $g(1)/g(0)$ that uses established scaling relations for the number of self-avoiding walks and the number of self-avoiding loops [2]. This gives

$$\begin{aligned} g(1) &= \sum_{\{i,j\}} \omega_{\rm walk}(L-1) + \sum_{i,j} \omega_{\rm loop}(|i-j|, L), \\ \omega_{\rm walk}(N) &= A_{\rm walk}\mu^{N-1}(N-1)^{\gamma-1}, \\ \omega_{\rm loop}(N, L) &= \omega_{\rm walk}(L-N) A_{\rm loop}\mu^N N^{-3\nu}, \end{aligned} \tag{S1L}$$

where $\omega_{\rm walk}(L-1)$ is the number of self-avoiding walks when a polymer of length $L$ forms a contiguous bond (shortening it by 1 monomer), and $\omega_{\rm loop}(N, L)$ counts the number of self-avoiding loops of length $N$. We model the entropy of the polymer outside the loop as a self-avoiding walk of length $L - N$. The sums are over all possible contiguous bonds and loops, which depend on the compatibility of motifs $i$ and $j$. The exponents $\gamma = 1.157$ and $\nu = 0.588$ are universal, and $\mu = 10.037$ on the FCC lattice (this coefficient $\mu$, which is standard notation, is not to be confused with the chemical potential $\mu$ in our simulations). The scaling amplitudes $A_{\rm walk}$ and $A_{\rm loop}$ are not universal, so we determine their relative magnitude by fitting to $g(1)$ from the Monte Carlo $g(s)$ for a single sequence. With this one fitting parameter, we can rapidly evaluate $\Psi$ for new sequences with no additional simulations or calculations. Specifically, we perform a linear fit of $\Psi$ to $T_{\rm c}$ for the block sequences (Fig B) and obtain $T_{\rm c}$ for any new sequence from its $\Psi$ value. This procedure allows us to generate the $T_{\rm c}$ distribution in Fig. 3(d) in seconds. A Python script to calculate

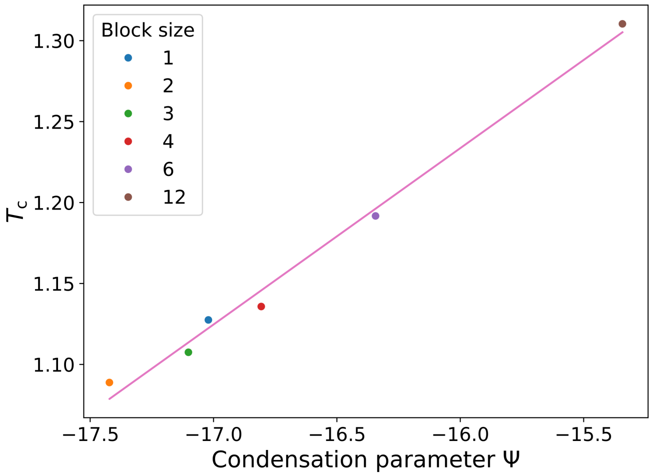


Figure B: The linear fit between $T_c$ from Monte Carlo simulations and $\Psi$ calculated via Eq. S1L. Slope=0.1089, intercept=2.9767.

$\Psi$ and $T_c$ for arbitrary sequences is available at https://github.com/BenjaminWeiner/motif-sequence/tree/master/condensation%20analysis.

# S1D Measuring viscosity in MD simulations

Monte Carlo simulations allow us to efficiently sample equilibrium phase properties by using non-physical moves to transition between states (e.g. polymer insertions and deletions). However, the expanded move set obscures dynamic properties such as viscosity. We were able to estimate viscosity in Fig. 4(c) of the main text by modeling the droplets as viscoelastic polymer melts, which led to Eq. 5 [3]. This analysis yielded the intriguing result that trans-bonds, rather than density, are the main source of elastic memory in our system. We wanted to test this result directly, so we performed molecular-dynamics (MD) simulations using the software package LAMMPS (see Molecular-dynamics methods below). In each simulation we fix the density and LAMMPS automatically calculates the pressure tensor $P_{ij}$, which is related to viscosity by the Green-Kubo formula [4]:

$$\eta = \frac{V}{k_B T} \int_0^\infty \langle P_{ij}(t_0) P_{ij}(t_0 + t) \rangle_{t_0} dt. \tag{S1M}$$

Fig C(a) shows the number of trans-bonds $\bar{t}$ versus density for $\ell = 2$ and $\ell = 12$ sequences, and Fig C(b) shows the viscosity. At each density, the $\ell = 12$ droplet has more trans-bonds, and this corresponds to a higher viscosity as predicted by Eq. 5. It follows that $\ell = 12$ droplets can be more viscous even when they are less dense than $\ell = 2$ droplets. These results bolster our conclusions from the main text: sequence primarily controls the material properties of the droplet via trans-bonds, which implies that large-domain sequences lead to more viscous droplets.

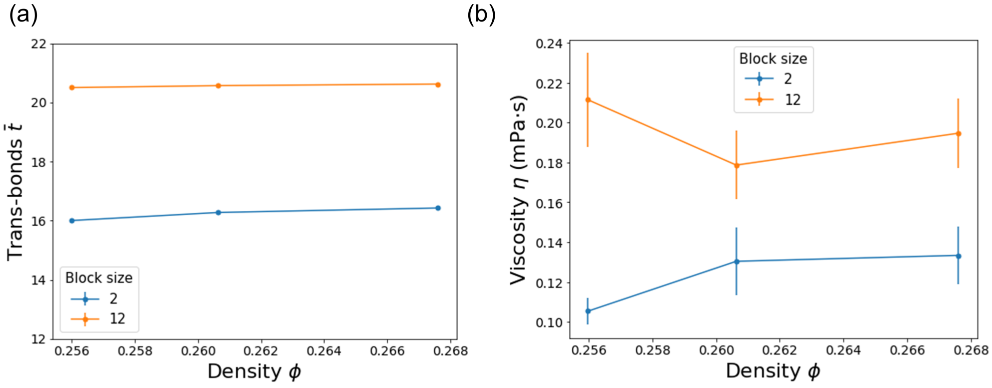


Figure C: In off-lattice MD simulations, large-domain sequences have more trans-bonds and a higher viscosity. All simulations performed at $T = 220$K, with $N = 4$ runs per density. (a) Trans-bonds $\bar{t}$ for the $\ell = 2$ and $\ell = 12$ sequences from MD simulations. Mean ± SEM across timesteps and across simulations. (b) Viscosity for the $\ell = 2$ and $\ell = 12$ sequences from MD simulations. Mean ± SEM across simulations.

## S1D.1 Molecular-dynamics methods

Coarse-grained MD simulations were performed using the LAMMPS software package [5]. Each simulation contained polymers of a single sequence at fixed number, volume, and temperature (fixed NVT). Densities were chosen to be higher than the right side of the binodal, so as to simulate a homogeneous dense system. Polymers were modeled as linear chains of spherical beads of radius $r_0 = 0.5$nm, where each bead represents a sticker domain "A" or "B." The stickers were connected by stretchable bonds given by

$$U_{\mathrm{b}}(r) = -\frac{1}{2} K R_0^2 \ln\left(1 - \frac{r^2}{R_0^2}\right), \tag{S1N}$$

where $R_0 = 5$ nm and $K = 0.56 k_{\mathrm{B}}T/\mathrm{nm}^2$. Specific bonds between motifs of different types were implemented through an attractive potential given by

$$U_{\mathrm{a}}(r) = \begin{cases} -\frac{1}{2} U_0 \left[1 + \cos(\frac{\pi r}{R_{\mathrm{c}}})\right], & r < R_{\mathrm{c}}, \\ 0, & r \geq R_{\mathrm{c}}, \end{cases} \tag{S1O}$$

where $U_0 = 4k_\mathrm{B}T$ and $R_\mathrm{c} = 2r_0 = 1$ nm. Motifs of the same type interacted through a shifted Lennard-Jones potential given by

$$U_\mathrm{r}(r) = 4\epsilon\left[\left(\frac{\sigma}{r}\right)^{12} - \left(\frac{\sigma}{r}\right)^{6}\right] + \epsilon, \tag{S1P}$$

where $\epsilon = 0.2k_\mathrm{B}T$ and $\sigma = 2^{5/6}r_0 = 2^{-1/6}$ nm= 0.89 nm, with a cutoff at $2r_0$ (the minimum of the potential). Between 550-575 polymers composed of 24 motifs were simulated in a 30×30×30 $\mathrm{nm}^3$ box with periodic boundary conditions at $T = 220$K, where the system was evolved according to Langevin dynamics. Initially, the attractive potential was set to zero by setting $U_0 = 0$ and the system was allowed to equilibrate for $1.5 \times 10^6$ timesteps of $\Delta t = 0.01$ ns. Then, $U_0$ was slowly brought up to the final value over the course of $1.5 \times 10^6$ time steps and the system was allowed to equilibrate for another $1.5 \times 10^7$ time steps. Afterwards, the position of each sticker and the pressure tensor were recorded over another $1.5 \times 10^7$ time steps. Fig D shows a snapshot of the simulation, where "A" and "B" motifs are rendered in red and blue.

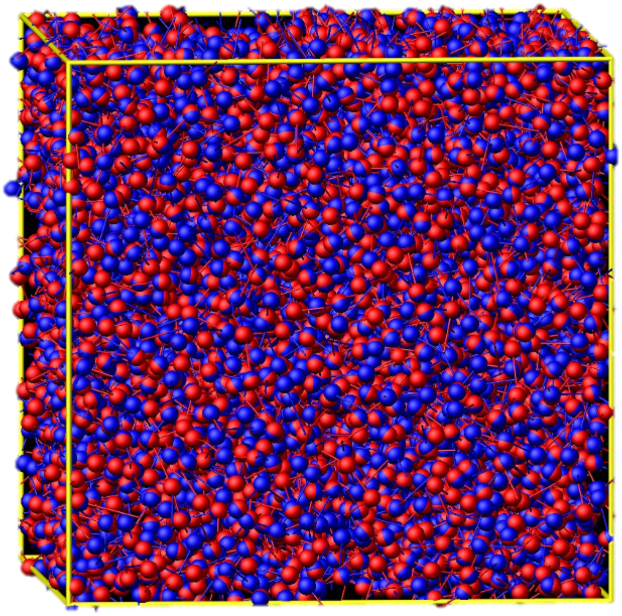

Figure D: Snapshot of a molecular dynamics simulation with the $\ell = 12$ sequence at temperature $T = 220$K, density $\phi = 0.268$.

## S1E Additional figures

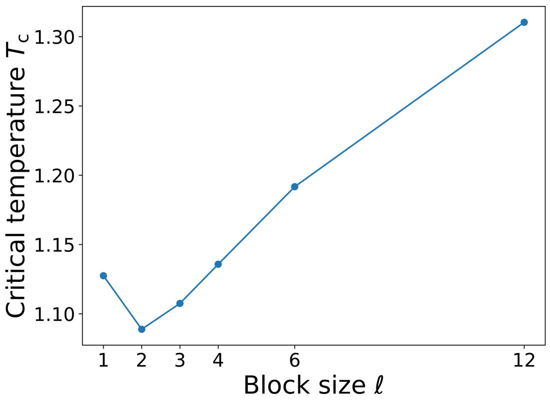


Figure E: The critical temperatures of $L = 24$ block sequences. $T_c$ is monotonic in block size $\ell$ except for the $\ell = 1$ sequence, which has strong trans-bond correlations (see Dense-phase correlations). Mean ± SD over three replicates. (Temperature uncertainties are too small to see.)

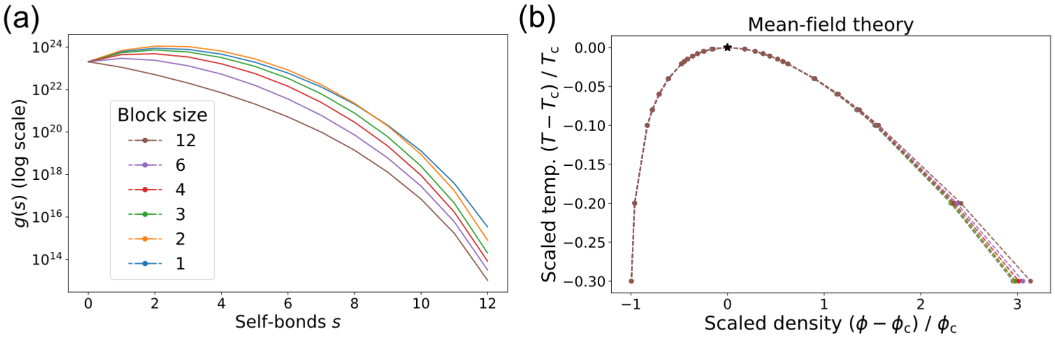


Figure F: (a) The density of states $g(s)$, i.e. the number of ways a given sequence can form $s$ bonds with itself, semi-log plot. Block sequences have large differences in $g(s)$ even for relatively rare states with large $s$. Block color code applies to all panels. (b) The phase diagram from the mean-field theory, rescaled by the critical temperature $T_c$ and critical density $\phi_c$.

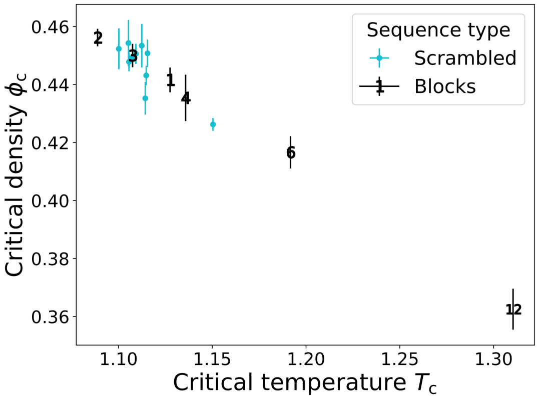


Figure G: Critical temperatures and critical densities of $L = 24$ block sequences and scrambled sequences, all with $a = b = 12$. For the block sequences, the plot markers denote block size $\ell$. Scrambled sequences cluster around the $\ell = 3$ block sequence, motivating the use of this sequence as the starting point for stoichiometry mutations in Fig. 3(b). Mean $\pm$ SD over three replicates. (Temperature uncertainties are too small to see.)

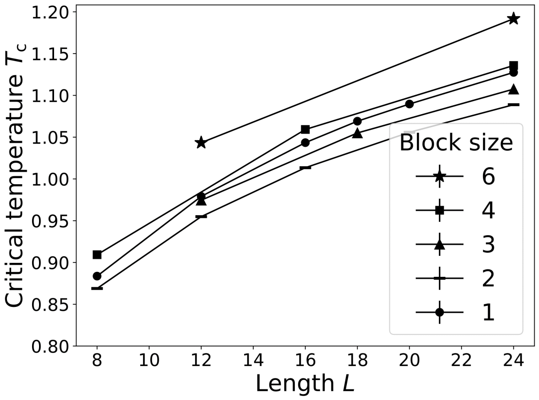


Figure H: $T_c$ as a function of length for sequences with different block sizes. Mean $\pm$ SD over three replicates. (Temperature uncertainties are too small to see.) The $T_c$ hierarchy is preserved across sequence lengths. Thus block size is a robust predictor of phase separation via its relationship with self-bond entropy.

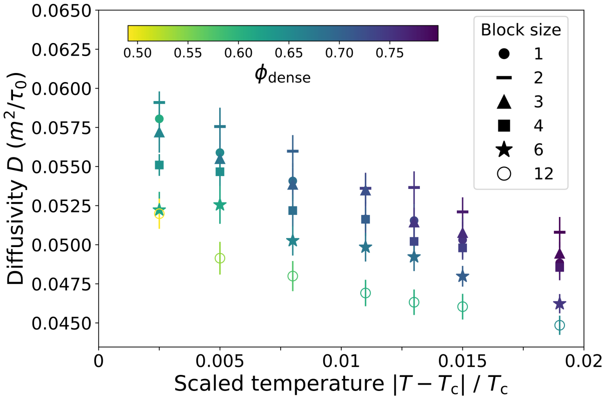


Figure I: Using the "Sticky Rouse Model" for unentangled polymer dynamics in a melt with cross-links [3], the dense-phase diffusivity $D = \frac{m^2}{\tau_b \bar{t}}$, where $m$ is the monomer size and $\tau_b = \tau_0 \exp(\beta\epsilon)$ is the bond lifetime, is plotted as a function of scaled temperature. For all sequences, lower temperatures correspond to higher densities and slower polymer diffusion. Importantly, the sequences with large block sizes and many trans-bonds (e.g. $\ell = 12$ and $\ell = 6$) have smaller $D$, in spite of their lower density. This coincides with the viscosity results in Fig. 4 of the main text, where the trans-bonds dominate the physical properties of the droplet. Color bar: droplet density.

# References

1. Semenov AN, Rubinstein M. Thermoreversible gelation in solutions of associative polymers. 1. Statics. Macromolecules. 1998;31(4):1373–1385.

2. De Gennes PG. Scaling concepts in polymer physics. Cornell University Press; 1979.

3. Rubinstein M, Semenov AN. Dynamics of entangled solutions of associating polymers. Macromolecules. 2001;34(4):1058–1068.

4. Hess B. Determining the shear viscosity of model liquids from molecular dynamics simulations. The Journal of chemical physics. 2002;116(1):209–217.

5. Plimpton S. Fast parallel algorithms for short-range molecular dynamics. Journal of computational physics. 1995;117(1):1–19.
